# Leser-Trélat Syndrome, an Underdiagnosed Cutaneous Paraneoplastic Syndrome That Could Aid in Early Detection of Colon Cancer: A Case Report

**DOI:** 10.7759/cureus.44907

**Published:** 2023-09-08

**Authors:** Oscar F Borja Montes, Tomas Escobar Gil, Camilo Cardona Solano, Daniel Padilla

**Affiliations:** 1 Internal Medicine, University of New Mexico School of Medicine, Albuquerque, USA; 2 Internal Medicine, Universidad Libre, Cali, COL; 3 Cardiology, University of New Mexico School of Medicine, Albuquerque, USA

**Keywords:** seborrheic keratosis, neoplasia, cancer, paraneoplastic syndromes, dermatology, oncology, adenocarcinoma

## Abstract

Leser-Trélat syndrome, a rare cutaneous paraneoplastic phenomenon, has gained attention for its potential role as an early indicator of underlying internal malignancies. This article presents a comprehensive case study of a 67-year-old male with a history of alcohol and tobacco use, emphasizing the importance of recognizing this syndrome in clinical practice. The sudden onset of seborrheic keratoses on the thorax and back, retrospectively identified as Leser-Trélat syndrome, prompted investigations that led to the early diagnosis of a colon adenocarcinoma. We discuss the pathophysiology, clinical relevance, and controversies surrounding this syndrome, highlighting the need for increased awareness among healthcare professionals. Timely recognition of Leser-Trélat syndrome can significantly impact patient care, leading to improved prognoses for associated neoplasms. This case underscores the importance of comprehensive evaluations and further research to enhance our understanding and management of cutaneous paraneoplastic syndromes.

## Introduction

Leser-Trélat syndrome is a paraneoplastic syndrome often associated with internal malignancies. It presents as a sudden increase in the size and number of seborrheic keratoses, primarily affecting the thorax and dorsum [1]. This syndrome is mainly linked to adenocarcinomas, predominantly located in the gastrointestinal (GI) tract. However, its association with aging might lead to an underestimation of its prognostic value, particularly when it indicates an underlying internal malignancy [2].

Currently, there is insufficient awareness and data regarding cutaneous paraneoplastic syndromes, resulting in potential underdiagnosis [2]. Recognizing these clinical manifestations could lead to the early detection of neoplasms and positively impact the overall prognosis [3]. In clinical practice, the identification and understanding of Leser-Trélat syndrome remain crucial for healthcare professionals [3]. The sudden and pronounced eruption of seborrheic keratoses should raise suspicion, especially in older patients or individuals with known risk factors for malignancies. Therefore, comprehensive evaluations, including a detailed medical history, physical examination, and appropriate imaging studies, should be conducted when this syndrome is suspected.

Additionally, better knowledge of the numerous cutaneous paraneoplastic syndromes among doctors should help with early detection and precise diagnosis, enabling earlier care [4]. Furthermore, ongoing research and collaborative efforts are necessary to unravel the underlying mechanisms linking this syndrome to malignancies, paving the way for potential targeted therapies and preventive strategies in the future.

## Case presentation

We present the case of a 67-year-old male with a medical history of hypertension, alcohol use disorder, and tobacco use disorder who presented to the emergency department due to a one-week history of black tarry stools and orthostasis. The patient had a significant alcohol intake of 10-12 beers daily. Two weeks prior, he had visited his primary care provider and was found to be hypertensive, for which he was prescribed an antihypertensive medication that he had not yet started. He had a routine colonoscopy approximately 10 years before, which was reported to be normal. However, he had never undergone an esophagogastroduodenoscopy (EGD).

**Figure 1:**
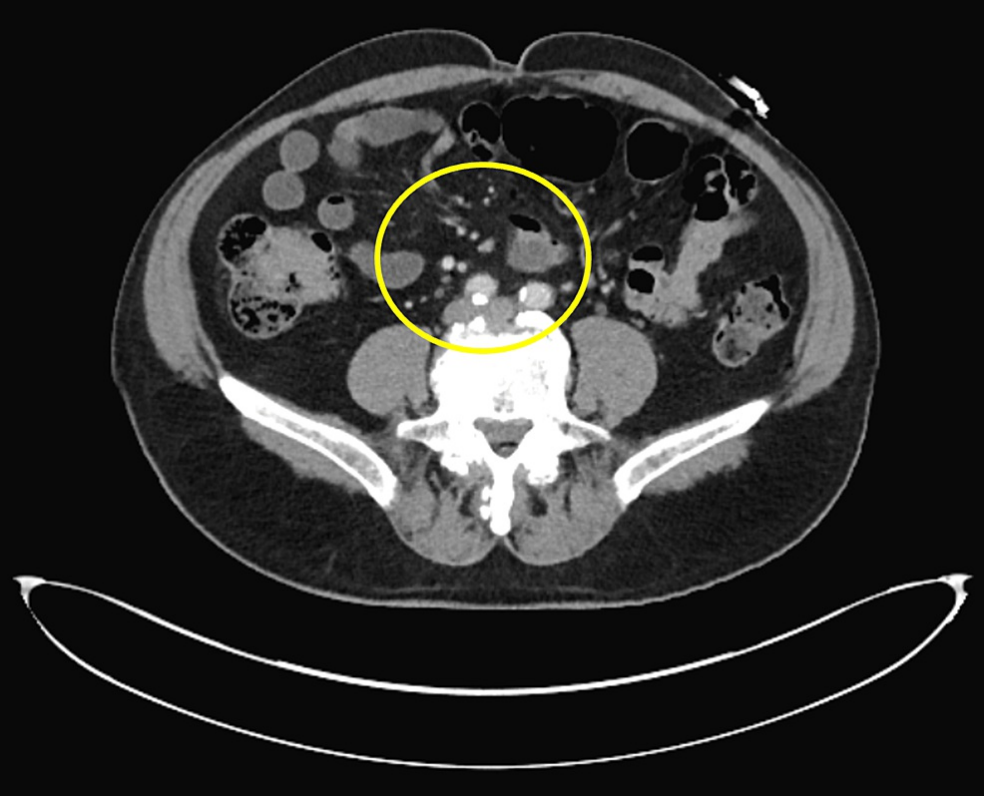
CT of the abdomen. Computed tomography shows nonspecific mild infiltration of the central mesenteric fat with an increased number of non-enlarged central mesenteric lymph nodes (see yellow circle).

**Figure 2:**
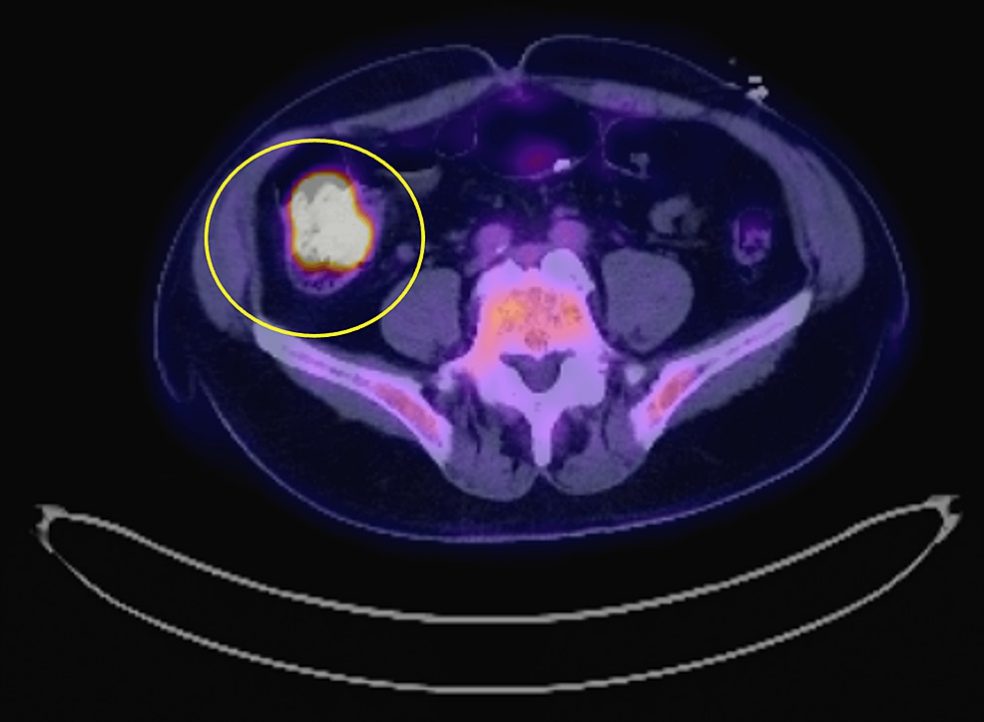
Fluorodeoxyglucose (FDG)-positron emission tomography (PET) of the abdomen. Hypermetabolic right colonic lesion of concern for malignancy (the involved area is marked with a yellow circle).

Upon physical examination, the patient was found to be tachycardic and mildly hypotensive. Laboratory tests revealed severe normocytic anemia with a hemoglobin level of 6.6 mg/dL, necessitating a blood transfusion of one unit of red blood cells (RBCs). He was initiated on intravenous proton pump inhibitor (PPI), selective bowel decontamination for spontaneous bacterial peritonitis (SBP) prophylaxis, and octreotide. An emergent EGD was performed, which reported segmental moderate inflammation with erythema in the gastric fundus. Subsequent colonoscopy revealed a 20-mm sessile polyp in the cecum. Biopsies were taken and indicated fragments of tubulovillous adenoma with high-grade dysplasia. An abdominal CT scan showed nonspecific mild infiltration of the central mesenteric fat with an increased number of non-enlarged central mesenteric lymph nodes (Figure 1). A PET scan also raised concern for malignancy due to a hypermetabolic right colonic lesion (Figure 2).

**Figure 3:**
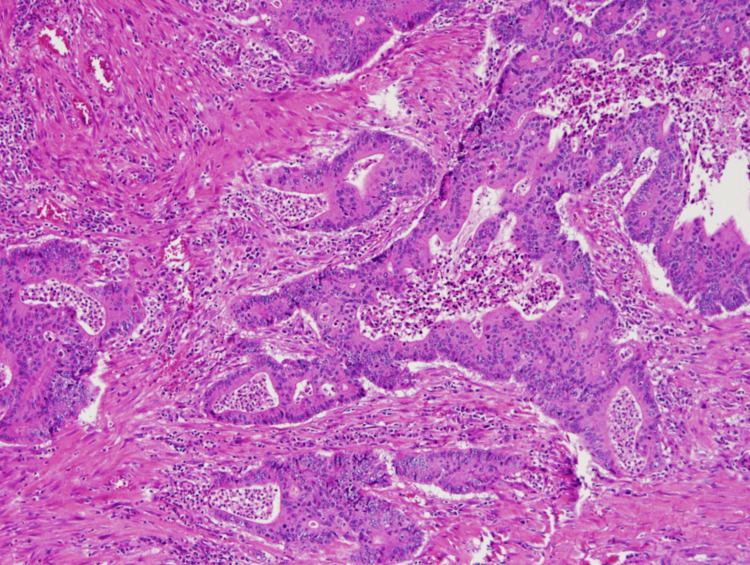
Biopsy of the ascending colon after hemicolectomy with 10x magnification. Moderately differentiated adenocarcinoma with invasion of the submucosa, arising from a tubulovillous adenoma.

Following evaluation, the patient was referred to General Surgery and Hematology Oncology, and a right hemicolectomy with ileocolic anastomosis was performed, with a biopsy revealing a moderately differentiated adenocarcinoma. The biopsy of the lesion can be seen in Figure 3.

**Figure 4:**
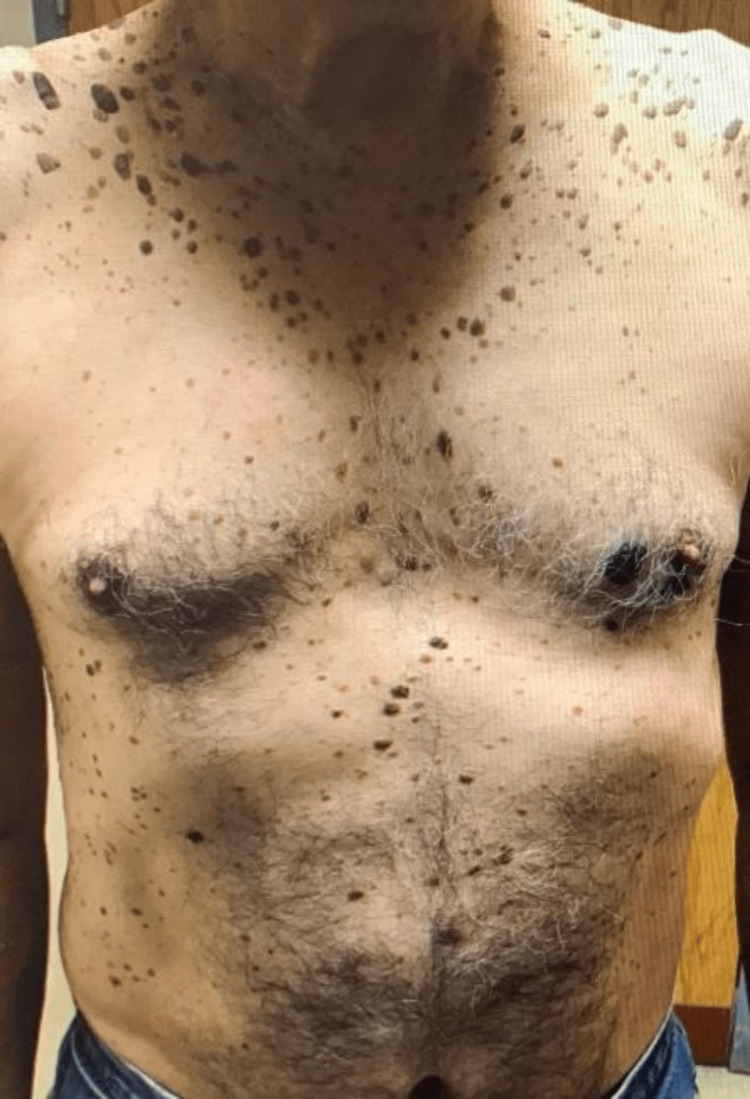
Multiple seborrheic keratoses on the trunk of a patient with a newly diagnosed adenocarcinoma of the colon. Evidence of multiple raised and pigmented plaques on the skin of the trunk. They vary in size and color, ranging from tan, brown, and black, and are slightly elevated with a stuck-on appearance. These lesions, in the setting of a newly diagnosed adenocarcinoma of the gastrointestinal tract, are consistent with Leser-Trélat syndrome.

**Figure 5:**
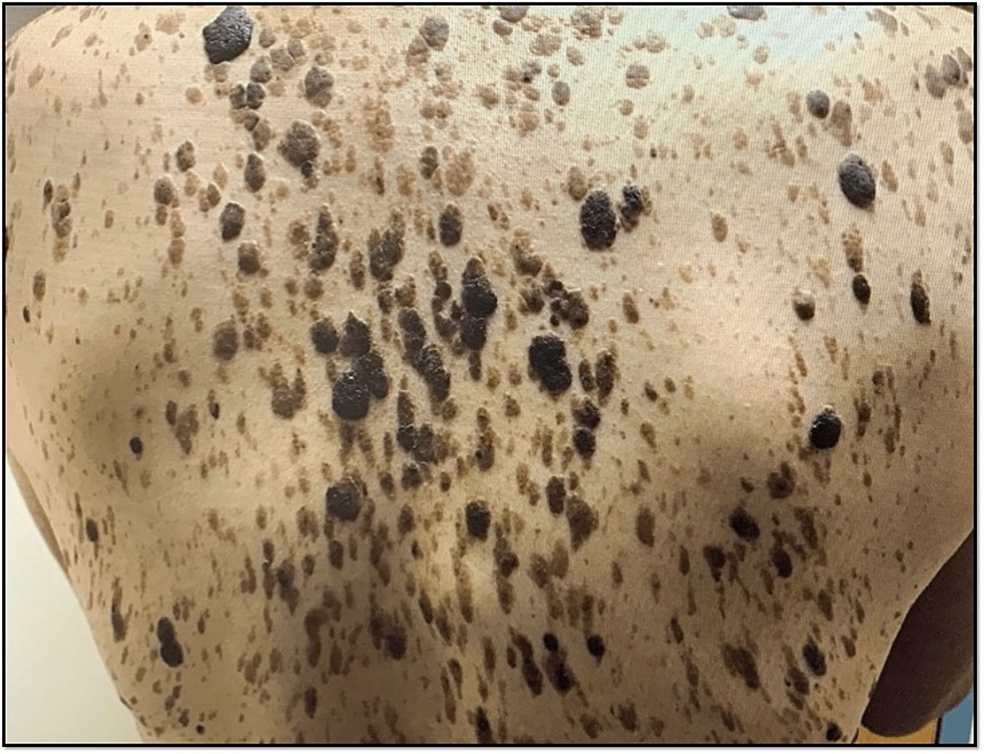
Multiple seborrheic keratoses on the trunk of a patient with a newly diagnosed adenocarcinoma of the colon. Evidence of numerous scaly, pigmented, stuck-on papules and plaques. They vary in size and color, ranging from tan, brown, and black, and are slightly elevated with a stuck-on appearance. These lesions, in the setting of a newly diagnosed adenocarcinoma of the gastrointestinal tract, are consistent with Leser-Trélat syndrome.

A few days after admission, the patient was observed to have multiple seborrheic keratoses lesions on the anterior thorax and back. Interestingly, there was no initial documentation regarding the acuity of onset of these skin lesions, and they were later retrospectively considered to be Leser-Trélat syndrome, a cutaneous paraneoplastic syndrome. The lesions can be seen in Figures 4, 5.

## Discussion

Leser-Trélat syndrome is a rare cutaneous paraneoplastic phenomenon often linked to internal malignancies, especially gastric adenocarcinomas, as well as other gastrointestinal malignancies, prostate cancer, and lymphomas [2]. Although its pathophysiology isn't fully elucidated, factors like human growth factor, EGF alpha, and EGFR are believed to contribute to seborrheic keratosis development [5]. Other theories propose tumor-derived epidermal growth factors as the culprits behind its clinical expressions [6].

A hallmark of Leser-Trélat syndrome is a sudden surge in the size and count of seborrheic keratoses on the thorax and dorsum. However, in older patients, the importance of this sign might be diminished due to the age-related occurrence of seborrheic keratoses [7]. Cutaneous paraneoplastic syndromes, Leser-Trélat syndrome included, are often underdiagnosed, causing potential delays in identifying underlying neoplasms. While the debate about its positive predictive value persists, the occurrence of Leser-Trélat syndrome in younger patients with confirmed neoplasms underscores its clinical relevance as an early predictor of internal malignancies [1-2].

In this case, the documented physical exam during admission did not detail any skin changes. The patient had an alcohol use disorder, a common contributor to various causes of GI bleeding, including esophageal varices and gastric or duodenal ulcers. Initial treatment did not address malignancy as a possible etiology. Subsequently, a computed tomography (CT) scan of the abdomen prompted a colonoscopy to assess for neoplastic disease.

On the other hand, it is important to state that the sign of Leser-Trélat is quite controversial [2]. The frequency of neoplasia and SKs increases in the elderly population, and its description is very loosely defined and not quantitative, so there are no standards for rigorously defining the sign [2]. Many studies have disputed its existence [2]. However, raising awareness and recognition of cutaneous paraneoplastic syndromes among healthcare providers can lead to timelier neoplasm detection [3]. This case report underscores the necessity for thorough evaluations and comprehensive investigations in suspected paraneoplastic syndrome cases, particularly when malignancy risk factors are present.

The early detection and management of paraneoplastic syndromes like Leser-Trélat syndrome can significantly influence patient care and impact treatment decisions for associated internal malignancies [3]. Further research and education on cutaneous paraneoplastic syndromes are pivotal for enhancing comprehension and management, ultimately leading to improved patient outcomes and heightened survival rates in underlying neoplasm cases.

## Conclusions

This case underscores the underdiagnosis of cutaneous paraneoplastic syndromes, like Leser-Trélat syndrome, in clinical practice. The positive predictive value of this clinical indicator is currently debated, largely due to the higher prevalence of seborrheic keratoses among the aging population. Nonetheless, the occurrence of this syndrome in younger patients with confirmed neoplasms lends weight to its significance as a meaningful clinical sign. Raising awareness among healthcare professionals about cutaneous paraneoplastic syndromes could facilitate the early identification of internal malignancies, ultimately leading to more prompt diagnoses.

Timely recognition and diagnosis of paraneoplastic syndromes, such as Leser-Trélat syndrome, play a pivotal role in enhancing patient care and potentially influencing treatment decisions. Practitioners should regard these skin manifestations as potential precursors for internal malignancies, especially when relevant risk factors are present. Further research and education in this domain are essential to deepening our comprehension and effective management of these clinical phenomena, ultimately resulting in improved patient outcomes and heightened survival rates in cases involving associated neoplasms.

## References

[1] Li M, Yang LJ, Zhu XH (2009). The Leser-Trélat sign is associated with nasopharyngeal carcinoma: case report and review of cases reported in China. Clin Exp Dermatol.

[2] Kirchberger MC (2019). Gastrointestinal: Eruptive seborrheic keratoses: sign of Leser-Trélat in gastric adenocarcinoma. J Gastroenterol Hepatol.

[3] Silva JA, Mesquita Kde C, Igreja AC (2013). Paraneoplastic cutaneous manifestations: concepts and updates. An Bras Dermatol.

[4] Wick MR, Patterson JW (2019). Cutaneous paraneoplastic syndromes. Semin Diagn Pathol.

[5] Mulero-Soto P, Sanchez-Vivaldi J, Rovira O, Arocho J, Pereira-Torrellas G, Martinez-Trabal J, Bolaños-Avila G (2022). Case report of Leser-Trelat sign as sequela of an atypical inflammatory process. Int J Surg Case Rep.

[6] Ponti G, Luppi G, Losi L, Giannetti A, Seidenari S (2010). Leser-Trélat syndrome in patients affected by six multiple metachronous primitive cancers. J Hematol Oncol.

[7] Sardon C, Dempsey T (2017). The Leser-Trélat sign. Cleve Clin J Med.

